# Incidence and predictors of post stroke seizure among adult stroke patients admitted at Felege Hiwot compressive specialized hospital, Bahir Dar, North West Ethiopia, 2021: a retrospective follow up study

**DOI:** 10.1186/s12883-023-03083-z

**Published:** 2023-01-25

**Authors:** Tadios Lidetu, Dagmawit Zewdu

**Affiliations:** 1 Department of Epidemiology and Biostatistics, College of Medicine and Health Science, Bahir Dar University, Bahir Dar, Ethiopia; 2 Department of Adult Health Nursing, College of Medicine and Health Science, Bahir Dar University, Bahir Dar, Ethiopia

**Keywords:** Incidence, Predictors, Stroke, Seizure, Bahir Dar, Ethiopia

## Abstract

**Background:**

A post stroke seizure is a period of neurological dysfunction caused by abnormal neuronal activity. Seizures after a stroke have an impact on patients' lives and increase mortality in stroke patients. It also has a negative impact on the prognosis of stroke. However, there is a scarcity of literature in Ethiopia on the occurrence of post-stroke seizures. Therefore, this study aimed to assess the incidence and predictors of post-stroke seizure at Felege Hiwot compressive specialized hospital, North West Ethiopia.

**Methods:**

An institution-based retrospective follow-up study was carried out at Felege Hiwot Compressive Specialized Hospital, North West Ethiopia from July 1, 2017 to June 30, 2021. The records of 568 stroke patients were reviewed using a random sample method. To find predictors of post-stroke seizures, we applied the log-binomial regression model.

**Result:**

The incidence of post-stroke seizures was 22.18%( 95% CI 18.83%—25.83%). Older age group (ARR = 2.49, 95% CI 1.33–4.69), hemorrhagic stroke (ARR = 1.99, 95% CI 1.25–3.17), surgical intervention (ARR = 1.85, 95% CI 1.22–2.81), and tramadol medication (ARR = 1.85, 95% CI 1.22–2.81) were found to be predictors of post stroke seizure.

**Conclusion:**

This study revealed that the incidence of post stroke seizure was high and older age, haemorrhagic type of stroke, surgical management, and use of tramadol anti-pain medication are risk factors for post-stroke seizures. Therefore, health care professionals must pay special attention and provide clinical care to patients who have risk factors for post-stroke seizure.

## Introduction

A seizure is defined as uncontrolled, abnormal electrical activity in the brain that can result in changes in consciousness, behavior, memory, or feelings [1]. Seizures may be either provoked or unprovoked. Provoked seizures, also known as acute symptomatic seizures, can occur as a result of electrolyte disturbances, acute toxic effects (antidepressants, sympathomimetic), Sepsis, CNS infections, traumatic brain injury, and stroke (ischemic or hemorrhagic) [1–3]. Seizures that occur after a stroke and have no prior history of epilepsy are referred to as post-stroke seizures [4]. Post stroke seizure (PSS) is a common and serious complication of stroke. It is classified as early or late seizure depending on when the seizure begins after the stroke [5].

The majority of seizures after a stroke are focal at first, but secondary generalization is common, especially in patients with late-onset seizures [4, 6]. The onset of post-stroke seizures is linked to a number of complex factors, including the type of stroke (ischemic or hemorrhagic), the location and size of the lesion, and the disease severity. Furthermore, high National Institutes of Health stroke scale score (NIHSS), significant cortical involvement, hypertension, sex, and alcoholism have all been linked to an increased risk of developing seizures [7–9].

Post stroke seizure affect patients’ lives and increase mortality in patients with stroke [7]. It also has a negative impact on stroke prognosis, including length of stay, disability rate, quality of life, physical and mental health of patients, as well as the burdens of in-hospital costs [9–11]. Evidence shows that post-stroke seizure is a common problem [12, 13]. Globally, 9% of stroke patients develop seizure [14]. In Africa, seizure after stroke varied considerably in different studies ranging from (9.3–14.9%) [11, 15, 16]. According to Ethiopian records, approximately 25% of stroke patients experienced post-stroke seizures [17].

More accurate knowledge on risk factors for PSS after the onset of seizure may have an impact on improving the prevention and treatment of PSS [7]. Seizures after a stroke are associated with increased hospitalization and mortality; therefore, seizure prevention and treatment are critical [18]. However, literature on the occurrence of seizures among stroke patients in Ethiopia is limited; we can confidently conclude that the incidence and predictors are not well established. As a result, this study aimed to assess the incidence and predictors of post-stroke seizures.

## Methods

### Study design and period

Institution-based retrospective follow-up study design was conducted using existing patient data (July/2017 to June/2021), with data extracted from September 20/2021 to October 10 / 2021.

### Setting and population

Patients admitted with stroke at Felege Hiwot comprehensive specialized Hospital (FHCSH) stroke care center from were included in this study. The hospital serves over 12 million people from the surrounding area and provides services to people in Amhara and neighboring regions. It is a referral hospital with over 400 hospital beds and a bed occupancy rate of 95.5% [19]. All hospitals which found in Amhara and neighboring regions refer stroke patients to FCSH for better diagnostic and management care. From July 2017 to June 2021, the hospital manages 2094 stroke patients. In the hospital (FHCSH) Stroke cases are diagnosed based on clinical presentation, physical examination, and imaging tests (CT scan and MRI). A post-stroke seizure is diagnosed when a patient has two or more seizures following a stroke and this is confirmed by physicians [19, 20].

### Inclusion and exclusion criteria

All stroke patients admitted at FHCSH from July 2017 to June 2021 were included in the study, whereas patients who had a seizure before admission, patients who died within 24 h of admission (because most early-onset seizures occur within the first 1 to 2 days after ischemia), and patient charts with missing data (outcome variable and/or ≥ two independent variables) were excluded. Twenty eight charts excluded due to missing data.

### Sample size determination

The total sample size was calculated using the double population-proportion formula with a 95% confidence level, 80% power, and a 1:1 ratio of unexposed to exposed groups. The final sample size was calculated using the previous study's variable nasogastric tube therapy (percentage of outcome in the unexposed group = 16.1%, percent of outcome in the exposed group = 25.5%, and Adjusted Risk ratio = 1.61). Finally, 568 patient charts were chosen at random from a total of 2052 patient charts. Fifty-two patients were excluded because they did not meet the study's eligibility criteria.

### Sampling procedure

Initially, the medical registration numbers of stroke patients admitted at FCSH stroke care center between July 2017 and June 2021 were collected. The patients' charts were then reviewed to determine whether they met the eligibility criteria or not. Following a review, 2052 patient charts met the eligibility criteria. Finally, 568 patient charts were chosen from a total of 2052 using a simple random sampling procedure (a computer-generated random sampling technique).


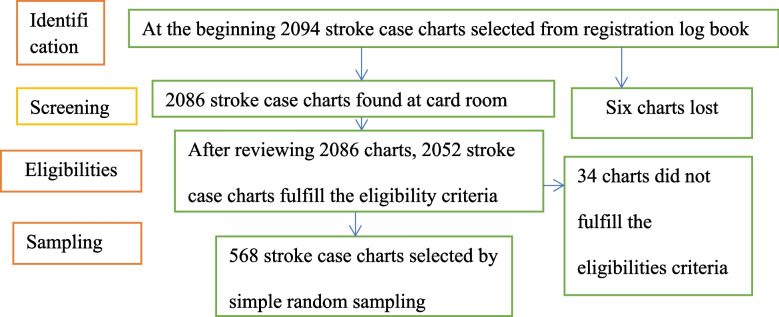


### Operational definition

#### Post-stroke seizure

Seizure is an episode of neurologic dysfunction caused by abnormal neuronal activity that occurs after a stroke without a previous history of epilepsy and stroked patient showed two episodes of seizure that confirmed by physician [21].

#### Stroke

It is sudden brain cell death due to lack of oxygen; the cause is vascular origin, and clinical presentations persist for more than 24 h [22].

#### Comorbidity diseases

A patient who had any disease in addition to stroke.

### Data collection tool and procedure

A structured checklist was used to collect the data. It was divided into sections that included socio-demographic variables, clinical variables, treatment-related variables, and seizure characteristics. The data were collected from existing medical records of stroke patients from July 2017 to June 2021, and the records were reviewed between September 20/2021 and October 10/ 2021.

### Data quality assurance

For the data quality assurance, a proper data extraction checklist was prepared. Before the actual data collection period, the consistency between the data extraction checklist and completeness of the recording system was checked using 5% of the sample size (28 charts). One-day training was given for the data collectors and supervisors. Finally, all the collected data were checked by the investigators for their completeness and consistency during the data entry, storage, management and analysis processes.

### Data management and analysis procedure

The checklist's completeness and consistency were manually checked. The data was entered using Epi data version 3.1 and then exported to Stata version-15 Statistical software for final analysis. The variance inflation factor for continuous independent variables and spearman's rank correlation for categorical independent variables were used to test for multicollinearity. The model goodness of fit was tested using Hosmer and Lemeshow's test, which yielded a value of 0.80. A log-binomial regression model with a 95% confidence level was used to assess the relationship between outcome and independent variables. Bi-variable and multi-variable log-binomial regression was used to identify significant predictors. In the bi-variable analysis, variables with *p*-values less than 0.25 were included in the multivariable analysis. A *P*-value of less than 0.05 at 95% confidence interval was used as a cut point in multi-variable analysis to declare a statistically significant association between predictors and post-stroke seizures.

## Results

### Socio-demographic characteristics

This study examined the charts of 568 adult stroke patients. There were 342 men (60.21%). The patients ranged in age from 23 to 115 years. The majority of patients, 407 (71.65%), came from rural areas (Table 1).

**Table 1:** Socio-demographic characteristics of patients with stroke at Western Amhara region, Ethiopia, 2021

Variables	Categories	Frequency	Percent
Sex	Female	226	39.79
	Male	342	60.21
Age	23 – 44	133	23.42
	45 – 64	127	22.36
	65 – 115	308	54.23
Residence	Urban	161	28.35
	Rural	407	71.65

### Clinical characteristics

Among 568 patients, 247(43.49%) had a Glasgow coma scale greater than twelve, and 483 (85.04%) patients had a unilateral stroke attack. The most common type of stroke (367 (64.61%) was ischemic (Table 2).

**Table 2:** Clinical characteristics of patients with stroke at Western Amhara region, Ethiopia, 2021

Variables	Categories	Frequency	Percent
GCS level	≤ 8	144	25.35
	9 – 12	177	31.16
	> 12	247	43.49
Stroked body part	Bilateral	85	14.96
	Unilateral	483	85.04
Type of stroke	Ischemic	367	64.61
	Hemorrhagic	201	35.39
Hypertension	No	268	47.18
	Yes	300	52.82
Heart diseases	No	474	83.45
	Yes	94	16.15
Diabetes mellitus	No	507	89.26
	Yes	61	10.74
GERD	No	339	59.68
	Yes	229	40.32
Dysphagia	No	324	57.04
	Yes	244	42.96

*GCS* Glasgow comma scale*, GERD* Gastro esophageal Disease

### Treatment-related characteristics

Five hundred forty-three patients (95.59%) were treated with non-surgical treatments (medication therapy). Tramadol was the most widely used anti-pain medication, accounting for 322 (56.69%). During the study period, less than one-third of the participants, 176 (30.99%), received oxygen therapy. In terms of hospital stay length, 397 (69.89%) patients stayed for three to seven days, while 171 (30.11%) stayed for eight to twenty-nine days (Table 3).

**Table 3:** Treatment-related characteristics of patients with stroke at Western Amhara region, Ethiopia, 2021

Variables	Categories	Frequency	Percent
Surgical intervention	No	543	95.59
	Yes	25	4.41
Mannitol	No	404	71.13
	Yes	164	28.87
Tramadol	No	246	43.31
	Yes	322	56.69
Metoclopramide	No	428	75.35
	Yes	140	24.65
Nasogastric tube insertion	No	312	54.93
	Yes	256	45.07
Oxygen administered	No	392	69.01
	Yes	176	30.99
Intravenous fluid therapy	No	383	67.43
	Yes	185	32.57
Length of hospital stay	3–7	397	69.89
	8–29	171	30.11

### Incidence of post stroke seizure

The incidence of seizure among stroke patients was 22.18% (95% CI 18.83%—25.83%).

### Predictors of seizure

Those variables with a *p*-value less than 0.25 on bi-variable log-binomial regression analysis were entered into multi-variable log-binomial regression analysis. In multi-variable log-binomial regression, four variables were significantly associated with the development of post-stroke Seizure (age, type of stroke, surgical intervention and tramadol treatment).

The risk of developing post-stroke seizures was 1.98 times higher in patients aged 45–64 versus patients aged 23–44 (ARR = 1.98, 95 percent CI 1.06—3.70) and the risk of developing a post-stroke seizure was 2.49 times higher in patients aged 65–115 versus patients aged 23–44 (ARR = 2.49, 95 percent CI 1.33–4.69). Patients with hemorrhagic stroke were 1.99 times more risk for post stroke seizure than those with ischemic stroke (ARR = 1.99, 95 percent CI 1.25–3.17). Patients who had surgical intervention were 1.85 times more risk for post-stroke seizure than those who did not have surgical intervention (ARR = 1.85, 95 percent CI 1.22–2.81). Patients who received anti-pain tramadol medication were 3.06 times more risk for post stroke seizure than those who did not receive anti-pain tramadol medication (ARR = 1.85, 95 percent CI 1.22–2.81) (Table 4).

**Table 4:** Bi-variable and multi-variable log-binomial regression analysis to the predictors of dysphagia among adult stroke patients at Western Amhara region, Ethiopia, 2021

Variables	Category	Seizure		CRR(95% CI)	ARR(95% CI)	***P***-value
		Yes	No			
Sex	Female	52	174	1	-	-
	Male	74	268	0.94 (0.69–1.29)	-	-
Age categorized	23–44	25	108	1	1	
	45–64	32	95	1.34 (0.84–2.13)	1.98(1.06–3.70)	0.032
	65–115	69	239	1.19 (0.79–1.79)	2.49(1.33–4.69)	0.005
Residence	Urban	46	115	1	1	
	Rural	80	327	0.69 (0.50–0.94)	0.64(0.46–1.90)	0.073
GCS level	≤ 8	43	101	1	1	
	9–12	42	135	0.79 (0.55–1.14)	1.54(0.94–2.54)	0.089
	> 12	41	206	0.56 (0.38–0.81)	0.89(0.60–1.32)	0.558
Stroked body part	Bilateral	35	50	1	1	
	Unilateral	91	392	0.46 (0.33–0.63)	0.80(0.46–1.42)	0.455
Type of stroke	Ischemic	50	317	1	1	
	Hemorrhagic	76	125	2.78 (2.03–3.79)	1.99(1.25–3.17)	0.004
Hypertension	No	52	216	1	1	
	Yes	74	226	1.27 (0.93–1.74)	0.90(0.61–1.32)	0.577
Heart diseases	No	101	373	1	-	-
	Yes	25	69	1.25 (0.86–1.82)	-	-
Diabetes mellitus	No	106	401	1	1	
	Yes	20	41	1.57 (1.10–2.33)	1.00(0.62–1.61)	0.989
GERD	No	22	317	1	1	
	Yes	104	125	6.99 (4.56–10.74)	4.35(0.54–5.47)	0.764
Dysphagia	No	31	293	1	1	
	Yes	95	149	4.07 (2.81–5.89)	2.94(0.99–4.34)	0.071
Surgical intervention	No	108	403	1	1	
	Yes	18	39	1.49 (0.98–2.27)	1.85(1.22–2.81)	0.012
Mannitol	No	70	334	1	1	
	Yes	56	108	1.97 (1.46–2.66)	0.94(0.54–1.63)	0.828
Tramadol	No	11	235	1	1	
	Yes	115	207	7.99 (4.39–14.50)	3.01(1.96–4.61)	< 0.001
Metoclopramide	No	99	329	1	-	-
	Yes	27	113	0.83 (0.57–1.22)	-	-
Naso-gastric tube	No	89	223	1	1	
	Yes	37	219	0.51 (0.36–0.72)	0.16(0.09–1.27)	0.061
Oxygen-therapy	No	84	308	1	-	-
	Yes	42	134	1.11 (0.80–1.54)	-	-
Intravenous fluid therapy	No	83	300	1	-	-
	Yes	43	142	1.07 (0.76–1.48)	-	-
Length of hospital stay	3–7	76	321	1	1	
	8–29	50	121	1.53 (1.12–2.08)	1.47(0.94–2.31)	0.092

*ARR* Adjusted Risk Ratio*, CI* Confidence Interval*, CRR* Crude Risk Ratio*, GCS* Glasgow coma scale*, GERD* Gastro esophageal Disease

## Discussion

The current study assessed the incidence and predictors of post-stroke seizures. The incidence of post-stroke seizures was 22.18% (95% CI: 18.83%—25.83%). This result was comparable to that of a study conducted in Addis Abeba (25%) [23]. This study had a higher incidence than studies conducted in Ghana (11.4%) [8], Italy (2.3%) [24], Singapore (6.64%) [6], and west Africa (14.9%) [25]. The possible reasons might be due to different post-stroke patient care (early hospitalization and using advanced device and therapy methods with specialized physicians in developed countries. Different socio-demographic status could also cause the discrepancy. Furthermore, the prevalence of comorbidity among stroke patients in this study high.

In this study, the risk of developing a post-stroke seizure was higher in patients aged 45–64 and 65–115 than in patients aged 15–44. This finding is consistent with research conducted in Japan and West Africa [26, 27]. This is due to the reason that old age stage is a peak period for developing epilepsy and seizures. However, new-onset seizures in the elderly are primarily the result of brain injuries and other secondary factors [28]. Besides that, physiological upset is common in the elderly and can result in acute symptomatic seizures, which are seizures that appear in close temporal association with a brain insult. Furthermore, several medications commonly prescribed to the elderly have been linked to hypernatremia, which can increase the risk of seizures. Another risk factor is excessive alcohol and recreational drug use [29].

Patients with hemorrhagic stroke were more risk for post stroke seizure than those with ischemic stroke. This finding is consistent with studies conducted in Egypt, Sudan, and India [15, 26, 30]. This is why patients with hemorrhagic stroke, particularly those involving the cerebral cortex, are more likely to develop seizures after their stroke. Furthermore, venous injury lesions are more likely to cause seizures because venous injury can affect the cortex [28]. Seizures following intracerebral haemorrhage may occur due to mechanical effects of the expanding haemorrhage of the cortex due to products of blood metabolism acutely and from hemosiderin depositions and gliotic scarring chronically [18].

Patients who underwent surgical intervention were more risk for post-stroke seizures than those who did not. This is consistent with studies conducted in Singapore and Saudi Arabia [31]. This is because the cause of seizures after surgery is a lack of oxygen reaching the brain, a condition known as hypoxia. Moreover Seizures following surgery are predicted by dialysis and brain damage [32]. Because cerebral vascular occlusion completely eliminates oxygen delivery to the brain, using supplemental oxygen, even when not hypoxic, appears to be a reasonable solution to try to correct the oxygen deficit [33].

Patients who received tramadol medication for anti-pain had a higher risk of developing post-stroke seizures than those who did not receive tramadol medication for anti-pain. This finding was consistent with studies conducted in the United States and Europe [34, 35]. This is because tramadol's inhibitory effects on serotonin and norepinephrine reuptake result in a distinct adverse effect profile, with two such adverse events being serotonin syndrome and seizures [36].

## Limitation of the study

Retrospective nature of the study design has its own limitation on assessing essential variables and exclusion of missed and incomplete data. Moreover, the severity of stroke is only assessed using GCS but not NHISS.

## Conclusion

This study showed that the incidence of post-stroke seizures was high [22.18% (18.83%—25.83%)]. Age, hemorrhagic type of stroke, surgical management of stroke, and tramadol use as an anti-pain medication have all been identified as risk factors for post-stroke seizure. As a result, in order to reduce the occurrence of post-stroke seizures, health care professionals should give special attention and clinical care to patients who have risk factors for post-stroke seizures. On top of that, the use of tramadol for stroke patients should be restricted, since it increases the risk of post stroke seizure.

## Data Availability

The data used to support the findings of this study are available from the following link (osf.io/6qtkw).

## Abbreviations

FCSHF: Felege Hiwot Comprehensive Specialized Hospital
IDR: Incidence Density Rate
GCS: Glasgow Coma Scale
LOHS: Length of Hospital Stay
MRN: Medical Registration Number
NGT: Nasogastric Tube
OPD: Out Patient Department
